# Current Trends in Neoantigen-Based Cancer Vaccines

**DOI:** 10.3390/ph16030392

**Published:** 2023-03-05

**Authors:** Szu-Ying Ho, Che-Mai Chang, Hsin-Ni Liao, Wan-Hsuan Chou, Chin-Lin Guo, Yun Yen, Yusuke Nakamura, Wei-Chiao Chang

**Affiliations:** 1 Department of Clinical Pharmacy, School of Pharmacy, Taipei Medical University, Taipei City 110, Taiwan; 2 Institute of Physics, Academia Sinica, Taipei City 115, Taiwan; 3 Ph.D. Program for Cancer Molecular Biology and Drug Discovery, College of Medical Science and Technology, Taipei Medical University, Taipei City 110, Taiwan; 4 Graduate Institute of Cancer Biology and Drug Discovery, College of Medical Science and Technology, Taipei Medical University, Taipei City 110, Taiwan; 5 TMU Research Center of Cancer Translational Medicine, Taipei Medical University, Taipei City 110, Taiwan; 6 Cancer Precision Medicine Center, Japanese Foundation for Cancer Research, Tokyo 135-8550, Japan; 7 National Institutes of Biomedical Innovation, Health and Nutrition, Osaka 567-0085, Japan; 8 Department of Medical Education and Research, Integrative Research Center for Critical Care, Wan-Fang Hospital, Taipei Medical University, Taipei City 116, Taiwan; 9 Department of Pharmacy, Wan Fang Hospital, Taipei Medical University, Taipei City 116, Taiwan

**Keywords:** cancer neoantigen, T-cell response, immune system, neoantigen vaccine

## Abstract

Cancer immunotherapies are treatments that use drugs or cells to activate patients’ own immune systems against cancer cells. Among them, cancer vaccines have recently been rapidly developed. Based on tumor-specific antigens referred to as neoantigens, these vaccines can be in various forms such as messenger (m)RNA and synthetic peptides to activate cytotoxic T cells and act with or without dendritic cells. Growing evidence suggests that neoantigen-based cancer vaccines possess a very promising future, yet the processes of immune recognition and activation to relay identification of a neoantigen through the histocompatibility complex (MHC) and T-cell receptor (TCR) remain unclear. Here, we describe features of neoantigens and the biological process of validating neoantigens, along with a discussion of recent progress in the scientific development and clinical applications of neoantigen-based cancer vaccines.

## 1. Introduction of Fundamental Properties of T-Cell Immunity

The human immune system acts like the military, with soldiers dedicated to identifying and attacking invaders such as foreign pathogens (viruses and bacteria) or dangerous cells in the body. There are two distinct forms of the human immune system: innate immune response and acquired immune response. Innate or nonspecific immunity is the first defense system that immediately responds to foreign pathogens and subsequently activates the acquired immune system. The innate immune response includes phagocytosis, mucosal barriers, inflammatory responses, a complement system, interferon secretion, and so on. The acquired immune response is defined as antigen-specific responses of the immune mechanism mediated by T and B cells. Particularly, T-cell immune responses play an important role in killing cancer cells. Thus, it is crucial to know the mechanism by which T-cell-mediated cancer immunity is activated [1].

Generated in the bone marrow, T cells are educated in the thymus, where some of them are eliminated by immune selection to avoid autoimmune responses. Immune selection occurs not only in the thymus, but also in the peripheral circulation and lymphatic systems, respectively referred to as central and peripheral tolerances. The development of central tolerance begins with negative selection, where T cells with a sufficient affinity to bind self-antigen major histocompatibility complex (MHC) molecules are eliminated. Before selection, T cells express both clusters of differentiation 4 (CD4) and 8 (CD8) molecules. After selection, they differentiate to maintain only CD4 or CD8 [2]. Selected and mature T cells then enter peripheral systems, where peripheral tolerance occurs to eliminate self-reactive T cells by means such as deletion, anergy, and suppression [3].

One major task of T cells in peripheral systems is to recognize antigens presented by MHC molecules on antigen-presenting cells (APCs), such as dendritic cells (DCs), macrophages, and B cells. This recognition in turn activates T cells, with CD4^+^ and CD8^+^ T cells respectively recognizing antigens presented by MHC class II and I molecules. For DCs, MHC class I molecules present endogenous or exogenous antigens, while MHC class II molecules primarily present exogenous antigens [4]. In the antigen-presenting process of MHC class I molecules in DCs, endogenous and exogenous proteins or peptides are trimmed from long fragments into short pieces through the proteasome and are translocated from the cytosol to the endoplasmic reticulum (ER), where they bind to MHC class I molecules to form peptide-MHC (pMHC) complexes. These complexes are then processed and transported to the cell surface to interact with T cells. In the antigen-presenting process of MHC class II molecules in DCs, extracellular peptides taken up by cells through phagocytosis or endocytosis undergo proteolysis and denaturation in the endocytic pathway, where they encounter MHC class II molecules that can recognize the motifs of short pieces of peptides. Interactions of these molecules allow the MHC class II-associated invariant chain peptide (CLIP) bound to antigen-presenting sites of MHC class II molecules to be hydrolyzed and replaced with the recognized fragments of peptides. pMHC complexes are then transported from the endocytic compartment to the cell surface for recognition by T cells. MHC class II-activated CD4^+^ T cells mainly release cytokines to recruit and activate more CD4^+^/CD8^+^ T cells and B cells, while MHC class I-activated CD8^+^ T cells can recognize non-self-antigens presented by MHC class I on virus-infected or tumor cells and secrete perforin and granzyme to kill these abnormal cells [1]. Most studies focus on CD8^+^ T cells, owing to the power of these cells to kill tumor cells directly. Nevertheless, CD8^+^ T cells are prone to tumor evasion when the cancer epitope presented by MHC class I is lost in tumor cells [5,6]. Moreover, the functions of CD4^+^ T cells are not only to activate more CD8^+^ T cells and B cells to kill tumor cells, but also to directly kill tumor cells through recognizing cancer antigens presented by MHC class II and releasing cytotoxic cytokines [7]. As such, antigen presentation by MHC molecules is crucial for T-cell immune responses against tumor cells [8].

## 2. Roles of Neoantigens in Immunotherapy

Neoantigens are derived from genetic aberrances in cancer cells, including chromosomal translocations, somatic point mutations, and insertions and deletions (indels) [9]. Most mutations occur in introns and some of them cause splicing errors. The mutated genes are transcribed and translated to produce mutated peptides, which are further hydrolyzed and displayed by MHC molecules for T-cell recognition that further elicit T-cell immune responses. The functional importance of cancer neoantigens has been attributed to three aspects: (1) tumor mutation burden (TMB) and neoantigens, (2) presentation of neoantigens by MHC, and (3) T-cell recognition of cancer neoantigens.

For the first aspect, the TMB is defined as the number of non-inherited mutations per million bases (Mb) of tumor genomic sequence measured by next generation sequencing (NGS) [10]. TMB is a source of neoantigens. Positive correlation was found between the TMB and the number of cancer neoantigens [11]. Several lines of evidence have indicated that high TMB levels associate with better responses to immune checkpoint inhibitor (ICI) therapy in a variety of tumors [12,13]. A clinical study refined this observation by dividing ICI-treated non-small cell lung carcinoma (NSCLC) patients based on their TMB levels (high, medium, and low TMBs). The progression-free survival (PFS) of the high-TMB group was found to be better than the other two groups (medium, and low TMBs) [14]. In addition, results from melanoma patients receiving adoptive T-cell therapy indicated that TMBs were associated with clinical benefits; patients with a higher TMB had longer progression-free survival (PFS) and overall survival (OS) than the rest [15]. Recent retrospective research also suggested that ICI monotherapy may be more effective in metastatic triple-negative breast cancer patients with a high TMB, regardless of the monotherapy or combination therapy received [16]. A higher TMB is thought to generate larger numbers of tumor-specific neoantigens, corresponding to a better chance to be recognized by multiple tumor-antigen-specific T cells. Therefore, tumor neoantigen burden (TNB) instead of TMB is proposed as a biomarker to predict the tumor immunogenicity [11]. Although a large number of neoantigens are considered to allow the immune system to recognize cancer cells and activate CD8^+^ T cells to attack cancer cells, there is no precise method to quantify the number of neoantigens presented by the MHC. It is also not clear how to link the correlation between the number of neoantigens and the strength of T-cell responses in a quantitative manner. Indeed, the immune conditions of patients are largely unknown, which makes predictions more complicated, as the number and activities of T cells among individuals can vary significantly. As such, large uncertainty remains in estimating patients’ responses to ICI treatment. The uncertainty mainly arises for two reasons. First, despite mutated peptides being trimmed into small fragments of neoantigens, not all neoantigens are recognized by T cells. Moreover, the strength of immune recognition does not completely rely on the number of neoantigens. Despite that mutated peptides are trimmed into small fragments of neoantigens, not all neoantigens can be presented on MHC and further recognized by T cells to elicit the following immune responses. Thus, a person with a smaller TMB might still exhibit stronger T-cell reactivity than the average [17]. Additionally, there are intrinsic variations in neoantigens, namely, a neoantigen might be universally expressed by all tumor cells as a clonal antigen or just by a subpopulation of tumor cells as a sub-clonal antigen [18,19]. The clonal antigen is able to elicit T-cell responses, whereas the sub-clonal antigen might fail to do so [20]. These variations within tumors result in the intra-tumor heterogeneity. If such heterogeneity of neoantigens is ignored, TMB or TNB-based predictions of responses to immunotherapies might be under- or overestimated.

The second aspect is the presentation of neoantigens by MHC. The formation of a neoantigen is influenced by several factors such as peptide splicing and cellular stress. Thus, the context of the neoantigen is variable among individuals [21]. Even if a neoantigen is generated, it is plausible that an effective immune response against tumor cells might not be elicited as there is another factor affecting cancer immune responses: MHC identification of the neoantigen. This factor can be affected by protein splicing, the transporter associated with antigen processing (TAP), peptide affinity, MHC molecule selectivity, and the genotypes of the human leukocyte antigen (HLA) [22,23,24]. Human HLA diversity leads to inter-tumor heterogeneity of the neoantigen profiles. Similarly, immune responses are affected by the stability and density of pMHC and the affinity of pMHC with the T-cell receptor (TCR) [25,26,27]. Indeed, diversity of the TCR repertoire is a critical factor in the neoantigen-mediated immune response, as each individual might respond differently to the same neoantigen. All these factors contribute to the uncertainty and complexity of the neoantigen-mediated cancer immune response.

For the third aspect, regarding T-cell recognition of cancer neoantigens, T cells can specifically recognize tumor neoantigens to mediate the neoantigen-specific T-cell responses against tumors. A meta-analysis showed a better clinical benefit in patients with high CD8^+^ tumor-infiltrating lymphocytes (TILs) after immunotherapy, regardless of cancer origins [28]. Results from a clinical trial of TILs in metastatic melanoma also showed a longer survival if the patients were with a higher frequency of neoepitope-reactive CD8^+^ T cells in their TIL adoptive cell therapy products [29]. Indeed, patients had more severe diseases when the patient-derived T cells failed to recognize a neoantigen epitope [30]. Conversely, cancer patients receiving immunotherapies exhibited increased neoantigen-specific T-cell responses. For example, Stevanović et al. reported that patients with a head-and-neck squamous cell carcinoma showed increased neoantigen-specific T-cell responses after receiving TIL therapy [31]. Consistent with the previous findings, anti-PD-L1 therapy showed better clinical outcomes in the metastatic urothelial carcinoma patients with early-stage neoantigen-reactive T-cell responses [32]. In parallel, engineered cancer neoantigen-specific T-cell responses have shown promising results against tumors. For example, improved therapeutic efficacy was observed in mouse models by administering a neoantigen vaccine [33,34]. Pipelines to generate neoantigen-specific TCR-T cells from patients’ TILs were also proposed and shown to elicit T-cell cytotoxicity and anti-tumor immunity in a xenograft mouse model for head-and-neck cancer [35]. Finally, there is an ongoing first-in-human phase I/II clinical trial that investigated the effects of engineered TCR-T-targeting neoantigens derived from hotspot mutations in a variety of solid tumors (NCT05194735). The results are expected in the near future.

## 3. Identification and Validation of Candidate Neoantigens

**Figure 1:**
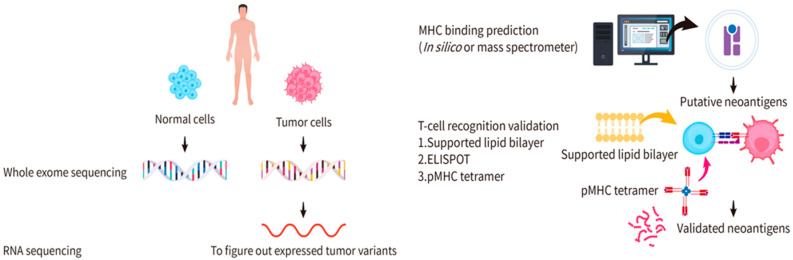
Identification of candidate cancer neoantigens and validation for functional neoantigens. Samples of both the normal and tumor cells from the same patient are collected for whole-exome DNA sequencing or RNA sequencing. Significant variants in protein-encoding regions and expressed mutations can be identified. Two general methods are applied to predict the MHC binding: in silico computational predictions and mass spectrum analyses. Enzyme-linked immunosorbent spot (ELISPOT), supported lipid bilayer (SLB)-based T-cell activation assays, and DNA barcode-based pMHC tetramer assays are used for functional validation.

To identify neoantigens, samples from both normal and tumor cells in the same patient are required. Sequencing is one of the techniques to figure out mutations of protein-encoding genes of an individual tumor. Whole-exome DNA sequencing and RNA sequencing are usually performed to identify mutations that are expressed [36,37]. Through whole-exome DNA sequencing on normal and tumor cells—one of the common techniques to detect non-synonymous somatic mutations—significant variants in protein-encoding regions can be identified [36]. RNA sequencing can further be used to identify the expressed mutations for more-precise predictions of potential neoantigens (Figure 1). Following these analyses is the functional verification of candidate neoantigens. Two important elements in this step are to determine: (1) whether the neoantigen can be recognized and presented by the MHC and (2) whether the pMHC can elicit a T-cell immune response. To this end, MHC identification of the antigen and TCR identification of the pMHC are often included in the functional verification of candidate neoantigens.

To verify whether an antigen can be presented by the MHC, two general methods are used: (1) in silico computational predictions and (2) mass spectrum analyses (Figure 1). Several software programs are available for in silico computational predictions, including Netchop, NetMHCpan, and IEDB [38,39,40,41]. The main principle of in silico computational predictions is to establish a predictive model through a database acquired by proteasome splicing, TAP channel selectivity, and epitopes through which the MHC molecules recognize the peptide. In comparison, mass spectrum analyses use known cancer neoantigens as a blueprint to compare and verify unknown neoantigens [42,43]. There are limitations of these two methods. In silico computational predictions have a high probability of false positives and inconsistent predictions from different software programs. However, mass spectrum analyses are expensive and suffer from the challenge that there is no robust reliable way to dissolve the neoantigens for analyses [44]. A high proportion of false negatives must be considered due to the incompleteness of the neoantigen repertoire [42].

To verify whether the pMHC can be recognized by the TCR to trigger T-cell activation, the current methods include enzyme-linked immunosorbent spot (ELISPOT), supported lipid bilayer (SLB)-based T-cell activation assays, and DNA barcode-based pMHC tetramer assays (Figure 1). The ELISPOT is a type of immunostaining assay that provides a quantitative measurement of cytokine release. This method involves pre-coating a plate with antibodies against cytokines released by T cells upon activation (e.g., anti-interleukin (IL)-2Ab), seeding the plate with T cells, and incubating T cells with the pMHC in each well. Cells are then removed and the medium is sequentially supplemented with biotinylated cytokine-specific detection antibodies, streptavidin-enzyme conjugates, and substrates for enzymes to create fluorescence. If the pMHC is recognized by the TCR, the cytokines released by the T cells are captured by the antibodies and the fluorescence created by the enzyme-substrate reaction can be detected. The extent of T-cell activation is justified by the depth of the fluorescence at the bottom of the plate [45,46]. A shortcoming of this method is that reagents are expensive for high-throughput screening. The SLB-based assay monitors interactions between the pMHC and the T cells. The pMHC-conjugated SLB is placed on a glass slide to allow for interactions with the T cells. The translocation of fluorescence-conjugated transcription factors, such as the nuclear factor of activated T cell (NFAT), upon T-cell activation is observed with an optical microscope (e.g., a total internal reflection fluorescent (TIRF) microscope) [47]. While this method offers direct observation of T-cell dynamics, its shortcoming is the difficulty of achieving high-throughput screening. In the DNA barcode-based assay, pMHC (a phycoerythrin (PE)-labeled multimerization backbone) and DNA barcode (with the DNA sequence of the pMHC peptide) are used to assemble pMHC tetramers [20,48], where PE is one of the most commonly used fluorescent dyes for flow cytometric analyses. The tetramers are mixed with T cells to allow for their interactions, followed by flow cytometry to sort out pMHC multimer-bound T cells. Because the signals are amplified by the DNA barcodes, the bound peptides can be easily detected. Nevertheless, this method has a shortcoming in that a direct observation of T-cell activation upon pMHC binding is unlikely.

**Figure 2:**
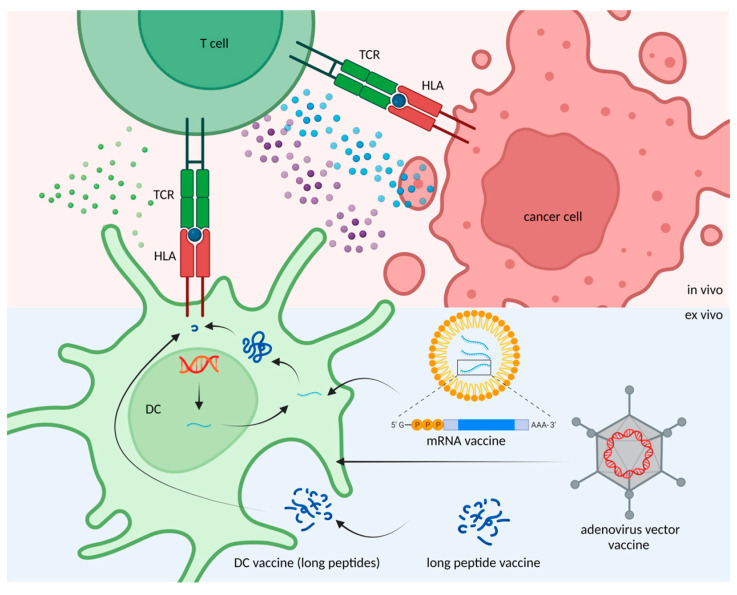
Methods for neoantigen delivery. Target DNA and mRNA molecules can be delivered by adenoviral vectors and liposomes to dendritic cells (DCs) for neoantigen production and presentation. Alternatively, neoantigens can be given in the form of peptides, which can be up taken by DCs in vivo.

Regardless of whether one is searching for candidate cancer neoantigens, screening the MHC-binding ability, or detecting T-cell activation, there are still many uncertainties that need to be resolved. Scientists expect to develop a comprehensive procedure to bring a glimmer of hope to cancer patients. This procedure fits the goal of personalized medicine, especially for cancer patients who have no other available options for treatment. Identifying and validating cancer neoantigens for cancer vaccines might target cancer cells in a precise manner to improve survival rates. To achieve the goal, different methods have been developed to deliver neoantigens as well as evoke T cell-responses (Figure 2), for example, mRNA (which encodes neoepitopes) can be encapsulated by liposomes for vaccine delivery. DNA-based neoantigen vaccine is delivered by adenoviral vectors through transfection. Direct injection of long peptide neoepitopes is another approach for neoantigen vaccination. To develop a safe, effective and efficient delivery strategy for neoantigen vaccines remains a challenge. In the following sections, we discuss the advantages, limitations, and the clinical discovery of neoantigen-based cancer vaccine therapy.

## 4. Application of Candidate Neoantigen Therapies

**Figure 3:**
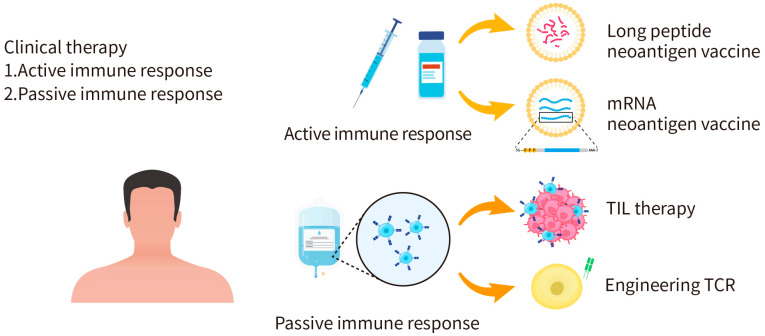
Applications of personalized cancer immunotherapy. Cancer immunotherapies can act through active immunity or passive immunity. The active immunity approach aims to activate the human immune system to attack tumor cells, such as the injection of long peptide or mRNA neoantigen vaccines. The passive immunity approach is to apply activated immune cells, antibodies, or cytokines to kill tumor cells.

In general, cancer immunotherapies act through active immunity or passive immunity (Figure 3). For example, T cells can be extracted or engineered to express neoantigen-specific T-cell receptors to kill tumor cells through passive immunity. In another approach, validated neoantigens can be injected into cancer patients in the form of a vaccine to elicit active immunity against tumors. By doing so, specific T-cell immune responses through MHC-mediated presentation of the neoantigen can be activated to attack specific tumor cells expressing the same neoantigen. The advantage of cancer vaccines is scalable and can be applied to different patients based on the personalized neoantigens. There are, however, limitations of neoantigen-based cancer vaccine therapies [49]. First, most of the cancer vaccine developments mainly focus on tumor-associated antigens (TAAs), which are self-antigens overexpressed by tumor cells; yet, T cells with high affinity for self-antigens are eliminated during T-cell development, which makes the vaccines less effective [50]. Although expression levels of TAAs are higher in tumor cells and lower in healthy cells, there is still a risk of off-target immune responses and toxicities. Nevertheless, the development of TAA-based cancer vaccines provides valuable experience and knowledge for the development of neoantigen-based cancer vaccines. Secondly, tumor cells often evade immune responses by losing the epitope [51]. Third, precise medicine has become a trend for cancer treatment. However, developing individualized cancer vaccines is expensive and time consuming, not to mention that the high efficiency delivery platform for cancer vaccines platform is still under development [52]. All these factors significantly impede the progress of neoantigen-based cancer vaccine therapies.

Besides these limitations, the design of cancer vaccines should consider two concerns. The first concern is whether the design should go for CD8^+^ or CD4^+^ T-cell responses. The second concern is whether the design should aim to induce new T-cell immune responses or boost preexisting T-cell immune responses. Of note, boosting the T-cell immune response indicates that cancer-killing T cells are already in the existing T-cell repertoire, yet previous data indicated that the expansion of preexisting T-cell populations could lead to decreased functional activity of immune cells [53]. Here, we update clinical trials of neoantigen cancer vaccines from 2019 to 2022 (Table 1). In earlier years, neoantigen vaccines were mainly applied on melanomas and glioblastomas. Recently, more patients and diverse cancer types were included in the trials, such as colorectal cancer (CRC), lung cancer, gastrointestinal (GI) cancer, and hepatocellular carcinoma (HCC). Peptides- and mRNA-based cancer vaccines are the major forms of design in the cancer vaccine immunotherapies.

The first neoantigen vaccine clinical trial (NCT01970358) tested a long peptide-based neoantigen vaccine (NeoVax) on melanoma patients [65]. In this trial, six untreated and high-risk (stage IIIB/C and IVM1a/b) patients were treated with five primary doses within the first four weeks and two booster doses in week 12 and 20, respectively. Each dose of NeoVax was composed of four peptide pools, which were injected separately at different sites of bodies. Results from the ELISPOT, intracellular cytokine staining (ICF), and flow cytometry demonstrated the immune responses elicited after vaccination, particularly the CD4-mediated T-cell responses. Moreover, none of the stage IIIB/C melanomas patients had a disease relapse 25 months after the regimen. Relapse was observed only in two patients enrolled with later stage (IVM1a/b) melanomas after the last dose of vaccination. Importantly, both of them achieved a complete radiographic response after subsequent treatment with four doses of the anti-programmed death 1 (PD-1) drug (pembrolizumab). In 2021, Hu et al. comprehensively reported the long-term follow-up (4 years) for these patients, during which another two other patients were enrolled in the trial. Three patients remained recurrence-free and another three relapsed but achieved a complete response after immunotherapy and surgery, while two patients developed unresectable metastatic tumors [66].It is noteworthy that the most common side effects were mild flu symptoms, discomfort at the injection site, rash, and fatigue. In addition, results from the clinical trial showed long-term maintenance of neoantigen-specific T-cell expansion and memory T cells after a long peptide-based vaccination. Indeed, a combination of a neoantigen vaccine and immune checkpoint blockers can be very helpful against metastatic tumors. Consistent with these findings, results from recent clinical trials have investigated the safety and efficacy of a long peptide-based neoantigen vaccine combined with immune checkpoint blockers in melanoma patients. For example, combination NEO-PV-01, a personalized cancer vaccine designed by BioNTech, with anti-PD-1 has been tested on melanoma [55,56]. Strikingly, the prolonged PFS of melanoma patients was strongly associated with increased clonal baseline TCR repertoires and longitudinal repertoire stability [56]. Thus, investigations of associations between neoantigen vaccine efficacy and immune repertoire characteristics might be an important research field in the future. The evaluation for safety and efficacy of using a long-peptide neoantigen vaccine in combination with immune checkpoint blockers is not an easy mission. Ellingsen et al. conducted a phase I/IIa trial with 12 patients to address this question. The clinical trial demonstrated an added benefit of long peptide-based neoantigen vaccines combined with anti-CTLA-4 monoclonal antibody ipilimumab in melanoma. The combination therapy was well-tolerated, with most adverse events were grade 1 or 2. Furthermore, the treatment response was observed in patients with any different baseline biomarkers of TMB, neoantigen burden, PD-L1 expression, lymphocyte tumor infiltration, or IFN-γ signature [62]. Based on the previous work on melanoma, NeoVax was tested on glioblastoma, which is considered as an immunologically “colder” tumor than melanoma [54]. Five doses of priming doses were given within the first month and two booster doses were given eight and sixteen weeks later. Still, the neoantigen vaccine was immunogenic and elicited neoantigen-specific T-cell responses with memory phenotypes. Moreover, the peripheral neoantigen-specific T cells migrated to the intracranial tumor site. Surprisingly, patients only experienced grade 1–2 side effects and the median PFS and OS were 7.6 and 16.8 months, respectively. This study demonstrated that the safety of a long peptide-based neoantigen vaccine and this approach is feasible for a “cold” tumor such as glioblastoma [59]. 

In addition to melanoma and glioblastoma, clinical trials of peptide-based neoantigen vaccines have been conducted in other cancer types, such as colorectal, lung, and liver cancers. For example, the Mycoryx trial was a phase I/IIa clinical trial (NCT01461148) enrolling 22 patients to evaluate the safety and immunogenicity of a frameshift peptide (FSP) neoantigen-based vaccine in DNA mismatch repair (MMR)-deficient CRC [57]. This vaccination was well tolerated by all patients and results demonstrated successful inductions of both humoral and cellular immune responses. Clinical responses from two of the three assessable patients showed stable disease as the best overall response; one heavily pretreated patient with bulky metastases showed stable disease and stable carcinoembryonic (CEA) levels for over 7 months. Besides CRC, neoantigen vaccines also offer a new therapeutic opportunity for lung cancer. Twelve heavily treated metastatic lung cancer patients received neoantigen peptide-pulsed autologous DC vaccines in a single-arm pilot study [59]. In this study, the objective effective rate was 25% and the disease control rate was 75%. The median PFS was 5.5 months and the median OS was 7.9 months. Despite the fact that none of the patients achieved complete tumor remission, this trial at least provided evidence of potential benefits for a neoantigen vaccine in advanced lung cancer patients. Combining a neoantigen vaccine and an immune checkpoint inhibitor has also been evaluated in lung cancer as well. NEO-PV-01, a long-peptide-based neoantigen vaccine, has shown vaccine-induced immunogenicity in combination with anti-PD-1 and chemotherapy as the first-line or later-line treatment for metastatic non-squamous NSCLC [55,63]. Furthermore, an epitope spread to non-vaccinating neoantigens was observed, indicating that a neoantigen vaccine can elicit a broader repertoire of immune response against the malignancies beyond the targets designed in the vaccine. Neoantigen vaccine therapy was also applied to HCC in a clinical study [60]. Two of the ten enrolled patients receiving long-peptide neoantigen vaccine remained relapse-free until the end of the trial, while the other eight patients experienced clinical relapses. The median relapse-free survival (RFS) of the 10 patients was 7.4 months since the first dose of vaccine received. Furthermore, 19.3 months of RFS was observed in the patients with neoantigen-induced T-cell responses, which was significantly longer than patients who had no responsive neoantigens.

Recently, promising results from the NOA-16 trial revealed great success of the IDH1R132H -specific peptide vaccine in glioma. The IDH1-vac is a peptide vaccine designed for the most common isocitrate dehydrogenase 1 (IDH1) mutation encoding IDH1R132H in gliomas. NOA-16 is a first-in-human, single-arm, open label, multicenter phase I trial to evaluate the safety, immunogenic responses, IDH1-specific T-cell responses, and efficacy of IDH1-vac. 33 patients with IDH1R132H gliomas were enrolled in the study. Vaccine immunogenicity was found in more than 90% of the patients, across patients with different MHC alleles. Furthermore, the adverse effects related to the vaccine were restricted to grade 1. In terms of the efficacy, 63% were progression-free and 84% were alive in a three-year follow-up [61]. With such encouraging results, a randomized phase I trial was initiated to test the IDH1-vac in combination with avelumab, an anti-PD-L1 drug, in the IDH1 mutant gliomas (NCT03893903) [67]. The clinical study is still ongoing.

In addition to peptide-based neoantigen vaccines, several groups have examined the clinical effects of mRNA-based neoantigen vaccines. In 2020, an mRNA neoantigen cancer vaccine was tested on four gastrointestinal (GI) cancer patients in a phase I/II clinical trial (NCI-18-C-0072) [58]. This mRNA vaccine contained 20 different types of epitopes, which were designed based on the mutant driver genes and the predicted-HLA-I-binding mutant peptides from each patient’s tumor. Two of the four patients were given a total of 0.13 mg and the other two were treated with 0.39 mg of the vaccine at 2-week intervals between the vaccinations. All four patients presented with grade 1 and 2 side effects, but no grade 3 or severe adverse events (SAEs) were observed. In total, 15.7% neoantigen-triggered specific T-cell responses were detected. Similar to NeoVax, CD4-mediated T-cell responses were observed more reactive than CD8-mediated ones. However, the epitope derived from autologous tumors was less responsive to specific T cells. Even so, this study at least supported the safety and potential clinical benefits of mRNA-based vaccines in cancer therapy. A currently published trial described a heterologous vaccine regimen, using the chimpanzee adenovirus (ChAd68) for priming and the self-amplifying mRNA (samRNA) for boosting, in combination with nivolumab and ipilimumab, to treat a variety of solid tumors, including metastatic MSS-CRC, non-small cell lung cancer (NSCLC), and gastroesophageal adenocarcinoma (GEA) [64]. In the interim analysis, no dose-limiting toxicities were observed. The regimen was found to be immunogenicity and can induce long-lasting neoantigen-specific cytotoxic T-cell responses. Furthermore, three out of seven (42.9%) of the MSS-CRC were alive at a 12-month follow-up. Further studies in the larger sample size are required to conclude the efficacy of this heterologous vaccine regimen in different solid tumors. As results of these approaches, a new design has been demonstrated in the neoantigen vaccination regimen by using different vectors to deliver the neoantigens for priming and boosting, portending a more diverse design of neoantigen vaccine regimens in the future.

There is a considerable body of published work of trials on neoantigen-based cancer vaccines. What lessons can we learn from these clinical trials? First, both long-peptide-based and mRNA-based vaccines can induce neoantigen-specific T-cell responses in different cancer types, yet only the T-cell response evoked by the long-peptide vaccine was shown to last several years. Second, among all the evoked T-cell responses, CD4-mediated ones were the most common effects after vaccination. Third, results from clinical trials revealed that side effects caused by both types of vaccines were relatively minor. Fourth, a combination of a neoantigen vaccine and ICI therapy should have possible clinical benefits for patients with metastatic tumors. Fifth, as most of those trials were in the phase I or phase II stage with limited patient sample sizes, clinical benefits still require further validation by larger-scale sample sizes. Taken together, what we learned from these clinical trials is a direction for vaccine design, such as the predictions of epitopes to induce CD4- or CD8-mediated T-cell responses. Increasing the precision of predictions and the selection of neoantigens should lead to stronger immune responses. Given that, the choice of cancer therapy is not only from existing anticancer drugs and ICIs but also from neoantigen-based cancer vaccines, a new option in the near future.

## 5. Challenges

Recently, spectacular progress has been made in the understanding of the mechanisms underlying immune evasion, the bioinformatic-based neoantigen prediction, and the clinical safety and efficacy of neoantigen-based cancer vaccines. However, challenges remain in the development of this type of vaccine for at least two reasons: (1) the limitation on the accessibility and (2) the nature of the tumor mutation. Clinically, the neoantigen-based cancer vaccines aim to target multiple tumor-specific mutations on a personalized basis. Through the next generation sequencing, thousands of mutations are often found in the tumor tissues of a single patient, however very few of them can eventually be identified as effective neoantigens. To identify the effectiveness of the neoantigen, two steps are widely used: (1) to analyze the physical interactions among the HLA molecules, the neoantigen peptides, and the T-cell receptors for the evaluation of immune recognition and (2) to confirm the immunogenicity of the neoantigen peptides by experimental validations before the vaccine preparation. Both steps are time-consuming, labor-intensive, and highly costly, making the pharmaceutical industry reluctant to develop the neoantigen-based cancer vaccine on a personalized basis. The cost mainly arises from the sequencing, the enzyme-linked immunosorbent spot assay (ELISpot), and the preparation of good manufacturing practice (GMP) grade materials. Despite the fact that the price for DNA and RNA sequencing has recently been reduced dramatically by pooling a large number of samples into a single sequencing run, this approach is not applicable for the development of personalized cancer vaccine as the vaccine is usually for a small sample, tailor-made, and for emergent use. Likewise, the functional validation of the cancer vaccine largely depends upon ELISpot analysis, which often requires the use of expensive reagents, a labor-intensive optimization of protocol, and a good quality control of the performance. The cost of good manufacturing practice (GMP) is another concern. GMP aims to ensure the quality of medicine products for human use, including the manufacture of neoantigen peptides, the typical duration of which is 4–6 weeks for a single peptide. As targeting multiple neoantigens within a single vaccine is the strategy to increase the successful rate in killing cancer cells [55,60,63], the cost and time consumption to make the GMP grade peptide vaccine can be tremendously high. All these factors limit the accessibility of the neoantigen-based cancer vaccine. How to conduct a high-throughput screening in a cost-effective and efficient way for cancer patients who are often in an urgent need is still a challenge to overcome. One potential way to resolve this issue is to use artificial intelligence and machine learning to improve the accuracy as well as efficiency of neoantigen identification. In any case, we should look forward to obtaining more stable and reliable screening platforms for the improvement of accessibility. The second concern for the limitation of the vaccines regards the nature of tumor heterogeneity. Cancers are known to have a high degree of heterogeneity and a capacity of metastasis, which cause the difficulties in cancer therapy. The tumor genome is also known to evolve constantly through genetic mutations, leading to the therapeutic resistance. Cancer vaccines are often designed by the neoantigens obtained from the primary tumor. It is likely that by the time the vaccine is ready, the tumor has already metastasized and mutated. In such case, vaccine is no longer suitable to fight against the tumor. In addition, the loss of HLA or the down-regulation of HLA expression is frequently observed in the tumor tissues, allowing cancer cells to evade the immune surveillance. In patients with such deleterious cancer genotypes or phenotypes, restoring normal HLA expression in cancer cells is of pivotal importance to evoke T-cell responses (i.e., transforming the “cold” tumor into a “hot” one). Without the restoration of HLA, the anti-tumor effect of the neoantigen-based cancer vaccine is limited.

## 6. Conclusions

Results from clinical trials have indicated an obvious effect of neoantigen-based cancer vaccines in cancer therapy. The side effects seem to be mild. With advancements in next generation sequencing and machine learning, the design for neoantigen cancer vaccines has become increasingly popular. Nevertheless, several limitations need to be resolved. These bottlenecks include effectively calculating the level of the neoantigen burden, quantitatively applying the neoantigen burden to predict the efficacy of cancer therapy, systematically monitoring T-cell immune responses of individual patients, establishing a reliable protocol to identify candidate neoantigens, and improving the clinical performance such as increasing survival rates and reducing adverse reactions. Combination therapies with immune checkpoint inhibitors provide other strategies to optimize the efficacy.

Cancer vaccine is personalized and unique to each patient. As such, the cost and the speed in vaccine development must be considered. Overcoming these limitations will rely on technological innovations in the future. Indeed, in order to gain more insights into these therapies, future studies should incorporate single-cell sequencing, artificial intelligence, and machine learning into the immunogenicity assessment as well as the design for usable neoantigen sequences. These techniques enable personalized neoantigen-targeting immunotherapies as a strong solution to fight against tumors.

## Figures and Tables

**Table 1:** Clinical trials of neoantigen vaccines from 2019 to 2022. The table summarizes the published year, vaccine name and vaccine type, enrolled patient number, targeted tumor types, efficacy and safety outcomes, and registry number of the trials.

Year	Vaccine	Vaccine Type	Patient Number	Study Phase	Tumor Type	Efficacy	Safety	Trial Identifier
2019 [54]	-	Long peptide	8	Phase Ib	Glioblastoma	Median progression-free survival: 7.6 months; overall survival: 16.8 months	Grade 1–2	NCT02287428
2020 [55]	NEO-PV-01	Long peptide	82 advanced melanoma (34), NSCLC (27), bladder cancer (21)	Phase Ib	Advanced melanoma, NSCLC, or bladder cancer	Treated following nivolumab, neoantigen-specific T-cell response was observed in all patients	Mostly Grade 1–2	NCT02897765
2020 [56]	NEO-PV-01	Long peptide	21	Phase I	Metastatic melanoma	Prolonged PFS is associated with increased clonal baseline TCR repertoires and longitudinal repertoire stability	-	NCT02897765
2020 [57]	Micoryx	Frameshift peptide (FSP) neoantigens (mutant AIM2, HT001, and TAF1B genes)	I (6) IIa (16)	Phase I/IIa	DNA mismatch repair (MMR)-deficient colorectal cancer	Vaccine-induced humoral and cellular immune responses were observed in all patients	Grade 1–2	NCT01461148
2020 [58]	mRNA-4650	mRNA vaccine	4	Phase I/II	Gastrointestinal cancer	15.7% of the potential neoantigens induced specific T cell immunity	Grade 1–2	NCT03480152
2021 [59]	Neo-DCVac	Dendritic cell vaccines (long peptide)	12	Phase I	Advanced lung cancer	Median progression-free survival: 5.5 months; overall survival: 7.9 months	Grade 1–2	ChiCTR-ONC-16009100, NCT02956551
2021 [60]	-	Long peptide	10	-	Hepatocellular carcinoma	Clinical relapse: 8 patients; relapse-free: 2 patients	Grade 1	ChiCTR1900020990
2021 [61]	IDH1-vac	Long peptide	32	Phase I	Gliomas with IDH1 mutation	Three-year progression-free rate: 0.63; death-free rate: 0.84. Patients with immune responses showed a 0.82 two-year progression-free rate of 0.82	Grade 1	NCT02454634
2022 [62]	UV1	Long peptide	12	Phase I/IIa	Unresectable metastatic melanoma	Treated in combination with ipilimumab, 91% of evaluable patients showed vaccine-specific immune responses. Clinical responses were observed in four patients (mPFS: 6.7 months, mOS: 66.3 months)	Grade 1–2	NCT02275416
2022 [63]	NEO-PV-01	Long peptide	38	Phase Ib	Metastatic non-squamous NSCLC	Treated in combination with anti-PD-1 and chemotherapy, de novo neoantigen-specific CD4^+^ and CD8^+^ T-cell responses were observed. Epitope spread to non-vaccinating neoantigens, including responses to KRAS G12C and G12V mutations.	Low grade	NCT03380871
2022 [64]	-	ChAd68 and samRNA	Fourteen metastatic MSS-CRC (7),GEA (6),NSCLC (1)	Phase 1/2	Metastatic MSS-CRC, non-small cell lung cancer (NSCLC) and gastroesophageal adenocarcinoma (GEA)	Vaccination combined with nivolumab and ipilimumab induced long-lasting neoantigen-specific CD8 T-cell responses. The median OS rate at 12 months was 8.7 months in MSS-CRC patients.	Mostly Grade 1–2	NCT03639714 (GRANITE)

PFS—progression free survival; OS—overall survival; mPFS—median progression free survival; mOS—median overall survival; TCR—T-cell receptor.

## Data Availability

Not applicable.
